# The Patient Journey in Interstitial Lung Disease: Mobility, Independence, and Psychological Burden

**DOI:** 10.3390/jcm14248697

**Published:** 2025-12-08

**Authors:** Ekaterina Krauss, Silke Tello, Daniel Kuhlewey, Poornima Mahavadi, Claudia Scharmer, Juergen Behr, Andreas Guenther, Gottfried Huss

**Affiliations:** 1 European IPF/ILD Registry & Biobank (eurIPFreg/bank, eurILDreg/bank), 35392 Giessen, Germany; 2 Center for Interstitial and Rare Lung Diseases (ZISL), Universities of Giessen and Marburg Lung Center (UGMLC), Justus-Liebig-University Giessen, German Center for Lung Research (DZL), 35392 Giessen, Germany; 3 Agaplesion Lung Clinic “Evangelisches Krankenhaus Mittelhessen”, Paul-Zipp Str. 171, 35398 Giessen, Germany; 4 Lungenfibrose e.V., Kupferdreher Str. 114, 45257 Essen, Germany; 5 Department of Medicine V, LMU University Hospital, Comprehensive Pneumology Center, Zentrum für Interstitielle und Seltene Lungenerkrankungen (ZISLE), German Center for Lung Research (DZL), LMU Munich, 81120 Munich, Germany; 6 Institute for Lung Health (ILH), 35392 Giessen, Germany; 7 Cardio-Pulmonary Institute (CPI), Klinikstr. 33, 35392 Giessen, Germany

**Keywords:** interstitial lung disease (ILD), idiopathic pulmonary fibrosis (IPF), health-related quality of life (HRQoL), European ILD registry (eurILDreg), European IPF registry (eurIPFreg)

## Abstract

**Background**: Interstitial lung diseases (ILDs) profoundly affect daily life, limiting mobility, independence, and emotional stability. While antifibrotic therapies may slow physiological decline, the living experience—characterized by breathlessness, cough, frailty, and psychological distress—remains insufficiently understood; this study therefore aimed to capture real-world patient perspectives on functional capacity, self-management, and mental health to identify treatable traits beyond conventional physiological measures. **Materials and Methods**: A cross-sectional quantitative online survey was conducted between September 2024 and January 2025 by Lungenfibrose e.V. in collaboration with the Center for Interstitial and Rare Lung Diseases (ZISL), Universities of Giessen and Marburg Lung Center (Giessen site). Patients with physician-confirmed ILD completed standardized instruments assessing dyspnea (MRC), cough intensity (VAS-Cough), frailty (CFS), and health-related quality of life (EQ-5D-5L). Data were analyzed descriptively across physical, functional, and psychosocial domains. **Results**: The majority of 69 respondents had idiopathic pulmonary fibrosis (64.7%) with a mean diagnostic delay of 1.4 ± 2.2 years; 69% were diagnosed within two years of symptom onset, and 77% were receiving antifibrotic therapy (nintedanib 57%, pirfenidone 19%). Functional limitations were substantial—55% were mobile for fewer than two hours per day, 73% reported mobility impairment, and oxygen use was common (51% during exertion, 26% at rest). Frailty increased over time (mean CFS 3.2 → 3.8), with 46% classified as fit, 36% vulnerable, and 18% frail. Dyspnea and cough remained burdensome (mean VAS-cough 40 ± 26; 58% moderate–severe), and health-related quality of life was reduced (mean EQ-VAS 56.5 ± 23.7), with high rates of anxiety/depression (78%), limitations in daily activities (76%), and pain/discomfort (74%). Despite overall satisfaction with care (mean 7.1 ± 2.5), respondents frequently reported unmet needs for psychological support and clearer communication about treatment and disease management. **Conclusions**: Despite antifibrotic therapy and structured specialist care, individuals living with ILD continue to face substantial physical and emotional challenges. Treatable traits—including frailty, dyspnea, inactivity, anxiety, and social isolation—emerge as key determinants of well-being. Multidisciplinary strategies integrating rehabilitation, psychosocial support, and patient education alongside pharmacological therapy are essential to preserve autonomy and improve quality of life in pulmonary fibrosis.

## 1. Introduction

Interstitial lung diseases (ILD) comprise a heterogeneous group of chronic, progressive disorders characterized by inflammation and fibrosis of the pulmonary interstitium [1]. Yet the clinical trajectory of ILD cannot be reduced to spirometric decline alone. Alongside measurable functional loss unfolds a patient journey—the living experience with the disease—that transforms movement, increases social seclusion, and disrupts the rhythms of everyday existence [2].

As respiratory capacity wanes, the act of breathing gradually becomes effortful, intruding on tasks once taken for granted. What begins as mild exertional dyspnea often progresses to a pervasive awareness of breathlessness that dictates the day. Patients describe shrinking physical horizons: social interactions and professional roles fade as exhaustion, cough, and dependence on oxygen redefine personal boundaries. This contraction of physical space is paralleled by an emotional narrowing—anxiety, uncertainty, and a quiet mourning for the loss of normality, once taken for granted [3]. These intertwined physical and psychological dimensions constitute the lived experience of ILD, revealing a disease burden that extends far beyond physiological indices [4].

Traditional outcome measures, such as forced vital capacity (FVC) or diffusing capacity (DLCO), capture disease progression but not the meaning of illness [5]. Patient-reported outcome measures (PROMs) have therefore emerged as indispensable instruments to describe how ILD affects daily life [6]. They quantify symptom intensity, mobility, self-sufficiency, and emotional well-being—domains that mirror how patients actually experience health and decline [7]. By translating subjective experience into structured data, PROMs reintroduce the patient’s voice into the scientific and clinical dialogue. They reveal what no pulmonary function curve can: how the disease feels, how it reshapes autonomy, and how individuals adapt to its persistent demands [8]. Integrating PROMs into ILD research enables a more authentic, human-centered understanding of treatment success—one that values stability of well-being as much as stability of lung function.

Although antifibrotic therapies such as pirfenidone and nintedanib have fundamentally altered the course of fibrotic ILDs by slowing decline in lung function and improving survival, the lived burden remains profound [9,10]. Many patients continue to face progressive breathlessness, physical dependency, and psychosocial distress despite optimized medical therapy, underscoring the need for early integration of supportive and even palliative approaches that address symptom relief, emotional well-being, and quality of life alongside disease-modifying treatment [11,12,13]. The persistence of these challenges underscores a critical unmet need in ILD care: improvement not only in how patients survive but also in how they live [14,15].

In Europe, the living experience of ILDs remains insufficiently explored, yet understanding how individuals adapt to breathlessness, navigate mobility loss, and preserve autonomy and dignity amid progressive decline is essential for developing compassionate and effective models of care [16].

Patient support groups have emerged as an essential complement to formal treatment, providing emotional validation, shared coping strategies, and a sense of community that counteract isolation [17]. Peer networks might effectively address social and psychological needs that pharmacological therapy alone cannot meet. Embedding structured support programs within multidisciplinary ILD pathways would meaningfully enhance resilience, continuity of care, and overall well-being at low cost and high impact.

Lungenfibrose e.V. is the national German patient association dedicated to individuals affected by pulmonary fibrosis and their families. Established as a non-profit organization, it serves as a central platform for education, peer support, and advocacy, bridging the gap between patients, clinicians, and researchers. The association plays a pivotal role in disseminating evidence-based information about diagnosis, treatment, and self-management, while fostering community connection among patients.

Against this background, the present study aims to illuminate these dimensions by capturing real-world experiences of people living with pulmonary fibrosis, focusing on mobility, independence, and psychological burden as key components of patient-reported well-being.

## 2. Study Objectives

This study aims to provide a comprehensive description of the lived experience and patient-reported burden of pulmonary fibrosis, encompassing physical, functional, and psychosocial dimensions of daily life. Specifically, it seeks to assess limitations in mobility, physical performance, and quality of life using validated PROMs and to identify unmet needs not captured by conventional clinical parameters.

## 3. Materials and Methods

### 3.1. Study Design and Framework

This study was conducted as an anonymous, cross-sectional, quantitative survey developed by the German patient association Lungenfibrose e.V. in collaboration with the Center for Interstitial and Rare Lung Diseases (ZISL), Universities of Giessen and Marburg Lung Center (UGMLC), and Justus Liebig University Giessen. The initiative was embedded within the eurILDreg framework [18].

### 3.2. Survey Platform

The survey was conducted between September 2024 and January 2025 among adults (≥18 years) with a physician-confirmed diagnosis of ILD. Participation was voluntary, uncompensated, and fully anonymous. All procedures conformed to the principles of the Declaration of Helsinki. Lungenfibrose e.V. was instrumental in recruiting participants through its nationwide network, online channels, and member newsletter, aiming to enable broad participation across different regions and disease stages.

Data were collected using SurveyMonkey^®^ (Momentive Inc., San Mateo, CA, USA), a General Data Protection Regulation (GDPR)-compliant, web-based platform widely used for non-interventional and patient-experience studies in European healthcare research. The platform ensured that all responses from EU participants were stored on EU-based servers in accordance with the GDPR and German data privacy law (DSGVO). Survey settings were configured to disable IP address logging, email tracking, and cookies, allowing for fully anonymous participation. A Data Processing Agreement (DPA) was established under the Enterprise plan to ensure contractual compliance with GDPR standards.

Participants received detailed information on data handling procedures and provided informed consent prior to participation, ensuring full transparency, data security, and participant privacy. Responses were encrypted, and no personal identifiers were recorded.

### 3.3. Survey Instruments and Variables

Although the organizing team aimed to capture as many aspects of the patient experience as possible, the survey length was deliberately limited to ensure feasibility for participants with respiratory impairment. The average completion time was targeted at approximately 15 min to encourage full participation and minimize fatigue-related dropouts. As a result, certain sociodemographic variables—such as age, comorbidities, smoking history, ethnicity, and educational level—were not included. This trade-off allowed for focused exploration of patient-reported symptoms, functional capacity, and quality-of-life measures while maintaining a manageable response burden for individuals living with pulmonary fibrosis. Also, it prioritized feasibility and accessibility for patients with significant physical limitations.

Participants self-identified as having a physician-confirmed diagnosis of ILD when completing the online questionnaire. Submission of medical documentation was optional to ensure full anonymity, as the survey focused on patient-reported experiences rather than clinical verification. Individuals were excluded if they were unable to complete an online survey or had previously undergone lung transplantation. No financial compensation was provided for participation.

The survey explored major aspects of the ILD patient journey, including symptom onset and diagnostic pathway, understanding of the disease and its management, current and prior treatment (antifibrotic therapy, oxygen use, pulmonary rehabilitation), and experiences or challenges related to antifibrotic medication. For untreated individuals, questions addressed reasons for non-initiation of therapy.

The questionnaire comprised both categorical and continuous items designed to capture disease-specific and patient-reported outcomes across domains representing physical, functional, and psychosocial aspects of disease burden.

#### 3.3.1. Mobility and Use of Assistive Devices

Questions addressed walking distance, daily physical activity, and the use of assistive devices such as rollators, wheelchairs, or oxygen systems. These data were used to quantify the degree of mobility limitation and dependency on support. Participants were asked: ‘On average, how many hours per day are you mobile, that is, on foot? Do you already use any of the following aids for household chores or leisure activities?’.

These data quantified the degree of mobility limitation and dependency on assistive support.

#### 3.3.2. Dyspnea and Physical Performance

The Medical Research Council (MRC) Dyspnea Scale (range 1–5) was used to grade the severity of breathlessness during routine activities, with higher scores indicating greater limitation in functional capacity [19].

#### 3.3.3. Cough Burden

The Visual Analog Scale for Cough (VAS-Cough) (0–100 mm) captured the self-perceived intensity and daily impact of coughing, a frequent and distressing symptom in fibrotic ILD [20,21].

#### 3.3.4. Frailty Status

The Clinical Frailty Scale (CFS) assessed overall physical fitness and vulnerability. This measure reflects functional reserve and general health status, particularly relevant in patients unable to attend tertiary ILD centers. The CFS scores patients on a scale from 1 (very fit) to 9 (terminally ill) and allows stratification into fit (CFS 1–3), vulnerable (CFS 4), and frail (CFS 5–9) categories [22,23]. The CFS can be assessed by healthcare professionals, caregivers or review of medical records [24,25,26]. The prognostic validity of the CFS in a diverse cohort of patients with fibrotic ILD has been demonstrated [27,28].

#### 3.3.5. Health-Related Quality of Life (HRQoL)

The EuroQol EQ-5D-5L instrument evaluated five domains—mobility, self-care, usual activities, pain/discomfort, and anxiety/depression—and included the EQ-VAS (0–100), a self-rated measure of overall health status [29,30].

### 3.4. Statistics

All analyses were performed using IBM SPSS Statistics (version 29, IBM Corp., Armonk, NY, USA) and Microsoft Excel (Microsoft Corp., Redmond, WA, USA). Given the exploratory, cross-sectional design of the survey, data analysis was descriptive in nature. Continuous variables were summarized as mean ± standard deviation (SD) or median [IQR], and categorical variables were reported as absolute frequencies (n) and percentages (%).

For the time-to-diagnosis variable, the interval between the onset of respiratory symptoms and the date of confirmed ILD diagnosis was calculated in days and subsequently converted to years for interpretability.

PROMs were analyzed according to established scoring frameworks. The MRC Dyspnea Scale (1–5) quantified exertional breathlessness, the Visual Analog Scale for Cough (VAS-Cough, 0–100) captured subjective cough intensity, and CFS (1–9) assessed overall fitness and vulnerability. CFS scores were further categorized into fit (1–3), vulnerable (4), and frail (≥5) to describe functional status distribution and longitudinal changes over five- and one-year intervals.

HRQoL was evaluated using the EQ-5D-5L descriptive system, which includes five domains—mobility, self-care, usual activities, pain/discomfort, and anxiety/depression. Each domain was analyzed by the percentage of responses within each severity level. The accompanying EQ-VAS (0–100) was reported as mean ± SD and categorized into quartiles representing poor (0–25), fair (26–50), good (51–75), and very good (≥76) self-rated health.

Ordinal variables, including satisfaction, activity limitation, and cough intensity, were categorized into quartiles to illustrate graded levels of impairment (minimal, mild, moderate, severe). Diagnostic delay and treatment initiation timelines were based on patient-reported dates, consistent with the survey’s anonymous design. Descriptive comparisons of CFS scores at three time points—five years before the survey, one year prior, and at the time of participation—were used to visualize longitudinal changes in physical vulnerability. CFS values for the time points ‘five years prior’ and ‘one year prior’ were retrospective self-assessments obtained at the time of the survey and were used to illustrate perceived functional decline rather than prospectively collected measurements. Consistent with the exploratory design of the study, no formal inferential testing was performed.

## 4. Results

A total of 213 individuals accessed the online survey, and 69 respondents (the response rate was 33%) completed all sections and were included in the final analysis. The survey was conducted in German and was available to patients across Western Europe. Most participants were from Germany (n = 58, 84%), while single participants were from Austria, Switzerland, and Finland (n = 1 each, 1.4%). The remaining participants reported “other” as their country of residence. All qualitative statements presented in this report were drawn verbatim from participant responses within the survey. The detailed survey is presented in Supplementary Materials.

Among respondents, the majority (64.7%) reported a diagnosis of idiopathic pulmonary fibrosis (IPF). Systemic Autoimmune Rheumatic Diseases (SARD)-associated ILD accounted for 13.7%, and chronic hypersensitivity pneumonitis for 9.8% of cases. A smaller proportion reported sarcoidosis (1.9%), unclassifiable ILD or nonspecific interstitial pneumonia (NSIP) (5.9%); 3.9% of participants were uncertain of their specific ILD subtype.

The interval between the onset of first respiratory symptoms and the date of confirmed ILD diagnosis varied widely among respondents. The mean time to diagnosis was 517 ± 788 days (median = 365 days, Q1 45.5–Q3 442 days), corresponding to 1.42 ± 2.16 years. Overall, 68.6% of participants received their diagnosis within two years of symptom onset, 17.6% reported delays of two to five years, and 13.7% experienced delays of more than five years.

**Figure 1:**
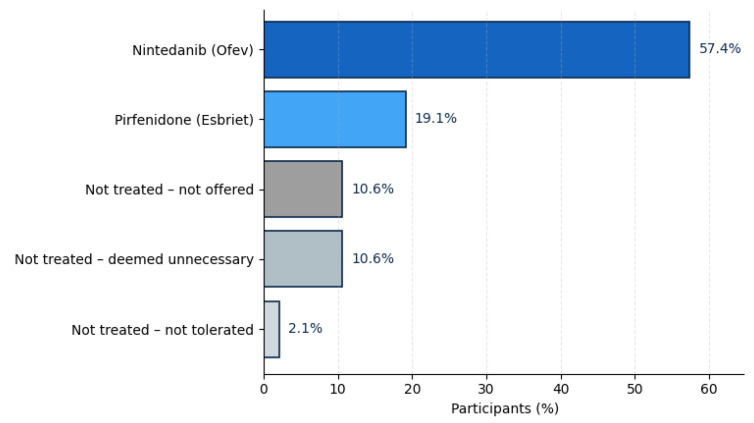
Distribution of antifibrotic therapies and reasons for non-treatment. Abbreviations: %—Percentage.

With regard to information provided on treatment, 76.6% reported current antifibrotic therapy, reflecting broad therapeutic uptake within the cohort. Most were treated with nintedanib (57.4%), while 19.1% received pirfenidone. The remaining 23.4% of participants were not on antifibrotic medication, most commonly because the therapy was not considered necessary by their physician (10.6%), not offered (10.6%), or discontinued due to side effects (2.1%). The data is presented in Figure 1.

On a 10-point satisfaction scale (1 = not at all satisfied, 10 = very satisfied), respondents expressed generally positive experiences with their medical care. The mean satisfaction score was 7.1 ± 2.5, indicating moderate to high satisfaction overall. Responses were skewed toward the upper range: 21.6% rated their care as 8, 19.6% each selected 5 or 9, and 15.7% gave the maximum rating (10 = very satisfied). Only 11.8% of participants reported low satisfaction (scores 1–3), suggesting that most patients perceived their treatment and care as adequate or good, though variation in individual experiences remained.

Overall, 35.3% of respondents reported being mobile for less than one hour per day, 19.6% for 1–2 h, and 23.5% for 2–4 h, while only 13.7% were active for 4–6 h and 7.8% for more than six hours daily, indicating that over half of participants (54.9%) were mobile for fewer than two hours per day.

As presented in Table 1, regular pulmonary rehabilitation, i.e., breathing therapy or physiotherapy sessions, was the most frequently reported measure (60.0%), followed by supplemental oxygen during exertion (51.4%) and use of a fingertip pulse oximeter (48.6%). A smaller proportion required oxygen at rest (25.7%), rollators (8.6%), or wheelchairs (5.7%), while only one participant (2.9%) reported use of non-invasive ventilation (NIV therapy). 11.4% of participants indicated that they currently required no assistive devices.

**Figure 2:**
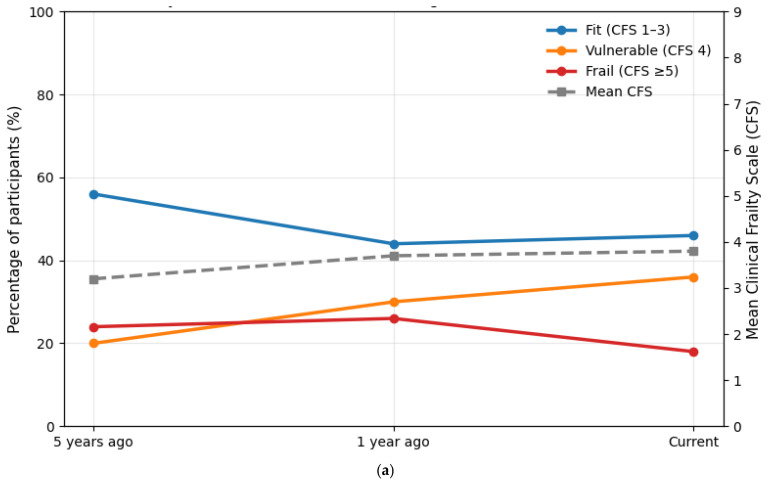

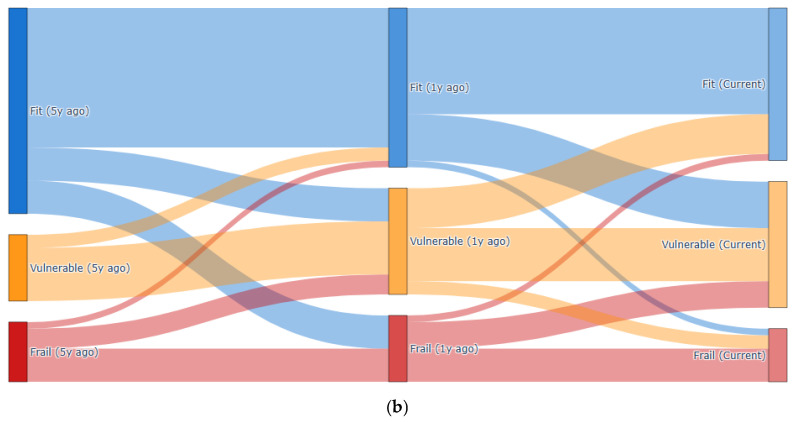
(**a**,**b**) Longitudinal self-reported transitions in CFS categories. Abbreviations: CFS—Clinical Frailty Scale.

In the CFS assessment, five years prior to the survey, 56% of respondents were classified as fit (CFS 1–3), 20% as vulnerable (CFS 4), and 24% as frail (CFS ≥ 5), with a mean CFS of 3.2 ± 1.8. One year before the survey, this distribution had already shifted toward higher frailty, with 44% fit, 30% vulnerable, and 26% frail (mean CFS 3.7 ± 1.4). At the time of the survey, the proportion of fit individuals had further declined to 46%, while 36% were classified as vulnerable and 18% as frail, corresponding to a mean CFS of 3.8 ± 1.2. Figure 2a,b show self-reported transitions in CFS categories.

**Figure 3:**
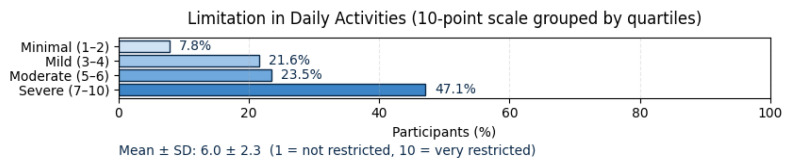
Limitations in daily activities.

Participants rated the degree of limitation in their daily activities (e.g., work, household tasks, leisure) on a 10-point scale (1 = not restricted at all, 10 = very restricted). The mean score was 6.0 ± 2.3, reflecting a moderate to high level of functional restriction across the cohort. When grouped into quartiles, 7.8% of respondents reported minimal limitation (scores 1–2), 21.6% described mild limitation (3–4), 23.5% indicated moderate limitation (5–6), and nearly half (47.1%) reported severe limitation (7–10), as presented in Figure 3.

**Figure 4:**
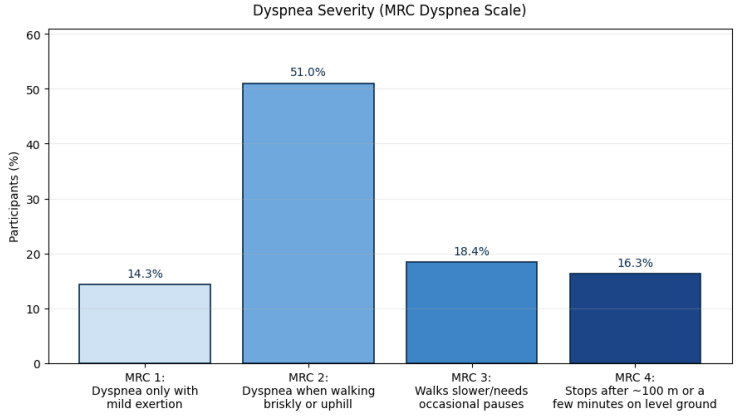
Dyspnea severity (MRC Dyspnea Scale). Abbreviations: MRC—Medical Research Council.

Dyspnea severity, assessed using the MRC Dyspnea Scale, showed that 51.0% of participants experienced shortness of breath when walking briskly or uphill (MRC grade 2), while 14.3% reported dyspnea only with mild exertion (MRC grade 1). An additional 18.4% walked slower than their peers or required occasional pauses due to breathlessness (MRC grade 3), and 16.3% needed frequent pauses after approximately 100 m or a few minutes of walking (MRC grade 4), indicating that nearly one-third of respondents experienced moderate to severe functional limitation (Figure 4).

**Figure 5:**
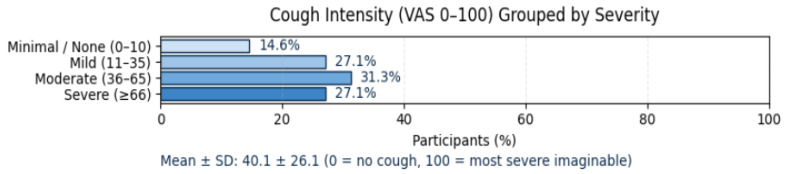
Cough intensity (Cough-VAS). Abbreviations: VAS—Visual Analog Scale.

Overall, the mean cough intensity was 40.1 ± 26.1, rated on a visual analog scale (VAS 0–100) where 0 = no cough and 100 = most severe cough imaginable; 14.6% of participants reported minimal or no cough (scores 0–10), 27.1% described mild cough (11–35), 31.3% experienced moderate cough intensity (36–65), and 27.1% indicated severe cough (≥66), demonstrating that over half of the cohort (58%) experienced at least moderate cough burden (Figure 5).

**Figure 6:**
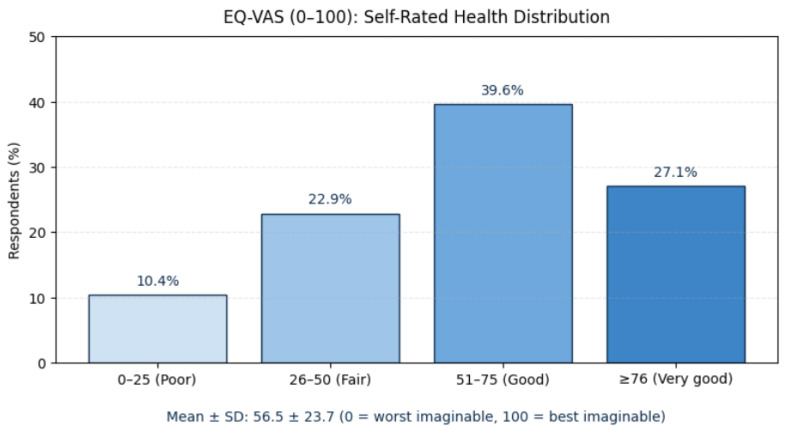
EQ-VAS: self-rated health distribution. Abbreviations: EQ-VAS—Euro QoL Visual Analog Scale, range 0–100, where 0 = worst imaginable health and 100 = best imaginable health; SD—Standard Deviation.

As part of the EQ-5D-5L instrument, which complements its five descriptive dimensions (mobility, self-care, usual activities, pain/discomfort, and anxiety/depression) to capture HRQoL from the patient’s perspective, self-rated health status assessed using the EQ-VAS (0–100) showed a mean score of 56.5 ± 23.7, with 10.4% of participants rating their health as poor (0–25), 22.9% as fair (26–50), 39.6% as good (51–75), and 27.1% as very good (≥76). The data is shown in Figure 6.

In the mobility domain, 72.5% of participants reported at least some degree of impairment: 23.5% had slight problems walking about, 39.2% experienced moderate problems, 9.8% reported severe problems, and 2.0% were unable to walk at all, while 25.5% indicated no mobility limitations.

For self-care, most respondents reported little to no difficulty: 70.6% had no problems, 17.7% reported slight problems, and 7.8% indicated moderate problems. Further, patients experienced greater limitations, with 2.0% reporting severe problems and another 2.0% stating they were unable to wash or dress themselves independently.

In the usual activities domain (e.g., work, household tasks, study, family, or leisure), 76.5% of respondents reported some degree of restriction: 25.5% described slight problems, 35.3% moderate, 13.7% severe, and 2.0% were unable to perform daily activities, while 23.5% reported full functional independence. Similarly, within the pain and physical discomfort dimension, 11.8% of participants reported no pain, whereas 39.0% experienced slight impairment, 39.2% moderate, and 9.8% severe pain or discomfort.

**Figure 7:**
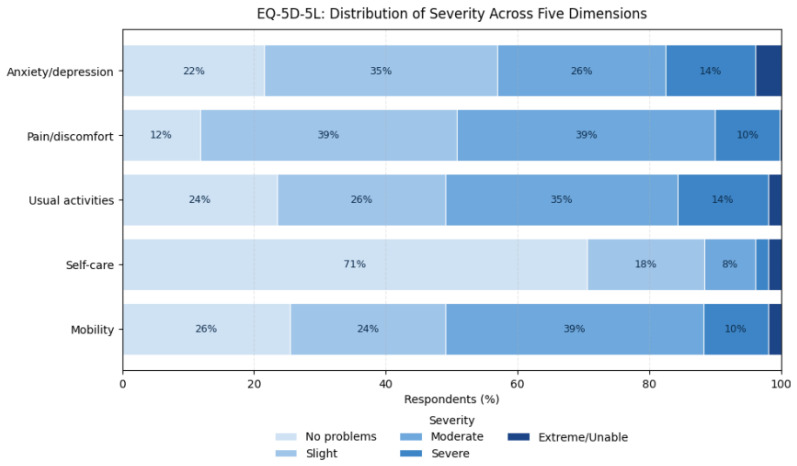
Distribution of responses across EQ-5D-5L domains. Abbreviations: EQ-5D-5L—European Quality of Life 5 Dimensions 5 Levels.

Finally, in terms of anxiety and depression, 78.4% of participants reported some degree of emotional distress—35.3% felt slightly anxious or depressed, 25.5% moderately, 13.7% very, and 3.9% extremely anxious or depressed—while 21.6% reported no symptoms. The data are presented in Figure 7.

## 5. Discussion

This study investigated the lived experience of individuals with ILD in Western Europe, with most respondents residing in Germany.

### 5.1. Diagnostic Delay and Treatment Uptake

The average interval between the onset of respiratory symptoms and a confirmed ILD diagnosis was approximately 1.4 years, though the range was wide, with over 13% experiencing delays beyond five years. This aligns with earlier European registry data and highlights persistent diagnostic gaps despite increased awareness and the expansion of ILD centers. Over three-quarters of respondents (77%) were receiving antifibrotic therapy—primarily nintedanib (57%) or pirfenidone (19%)—suggesting that access to modern treatment is comparatively well established within specialized networks. Nonetheless, one in four patients reported not receiving antifibrotic medication, underlining the ongoing variability in treatment pathways even within high-resource healthcare systems.

### 5.2. Mobility and Independence

Loss of mobility emerged as a dominant theme across patient-reported outcomes. More than half of participants were mobile for fewer than two hours per day, and 72% reported measurable impairment on the EQ-5D mobility dimension. One in ten patients faced severe mobility restrictions, and two percent were unable to walk at all. Assistive device use was common—over half required oxygen during exertion, and one-quarter needed oxygen at rest—while 60% reported participating in regular breathing or physiotherapy sessions. These findings depict a high dependency on physical support and rehabilitation measures.

### 5.3. Physical Performance, Dyspnea, and Cough

Dyspnea severity, quantified by the MRC Dyspnea Scale, revealed that over two-thirds of respondents experienced clinically significant exertional breathlessness (MRC ≥ 2), and nearly one-third were limited during everyday walking (MRC ≥ 3). The subjective burden of cough, measured by VAS, averaged 40 ± 26, with 58% reporting at least moderate cough intensity. Together, these symptoms not only compromise physical function but also affect social participation and mental health, reinforcing the multidimensional burden of ILD that extends well beyond spirometric decline.

### 5.4. Frailty and Functional Decline

Frailty trajectories demonstrated a gradual shift from physical robustness toward vulnerability over time. Five years before the survey, 56% of respondents were classified as fit and 24% as frail; currently, only 46% remained fit, while 36% were vulnerable and 18% frail. The mean CFS increased from 3.2 to 3.8, reflecting progressive functional decline despite therapeutic stabilization. This transition underscores that disease progression in pulmonary fibrosis is not only pulmonary but systemic—impacting endurance, resilience, and daily autonomy.

### 5.5. Health-Related Quality of Life

Across EQ-5D-5L domains, a consistent pattern of impairment was observed. More than three-quarters reported problems with mobility, usual activities, or pain/discomfort, and nearly 80% experienced some degree of anxiety or depression. Only one in five respondents described themselves as emotionally unaffected, illustrating the psychological toll of chronic breathlessness and uncertainty. The EQ-VAS mean of 56.5 ± 23.7 reflects moderate perceived health, with most patients rating their condition between “fair” and “good.”

### 5.6. Psychological Distress and Coping

Anxiety, frustration, and social isolation were common themes, consistent with data from the Graham et al. survey [31]. More than three-quarters of respondents reported emotional distress, and qualitative feedback emphasized fear of disease progression and loss of independence. Despite these challenges, many identified rehabilitation programs, family support, and patient associations as essential stabilizing factors.

### 5.7. Treatment Perceptions and Information Needs

Although overall satisfaction with medical care was high (mean 7.1/10), approximately 40% of participants expressed a desire for more comprehensive information about medication effects, prognosis, and rehabilitation options. This reflects the persistent tension between clinical efficacy and patient-perceived benefit. Transparent communication, shared decision-making, and continuity of care within specialized ILD centers were repeatedly highlighted as protective elements that foster trust and engagement.

### 5.8. Lungenfibrose e.V.

The collaboration with Lungenfibrose e.V. enabled participation across regions and disease stages, including individuals who may not be regularly seen in specialized ILD centers. Beyond facilitating recruitment, Lungenfibrose e.V. served as an important link between clinical care and the lived patient experience, providing guidance, emotional support, and advocacy. Its active involvement reflects the increasing integration of patient associations into ILD research and care, strengthening collaboration between patients, clinicians, and investigators.

Respondents frequently identified patient associations as essential sources of stability, empowerment, and psychosocial support. Our findings illustrate that pharmacologic progress alone does not resolve the enduring functional, emotional, and informational challenges faced by patients. While Europe’s integrated ILD networks and universal healthcare systems ensure broad access to antifibrotic therapy and rehabilitation, greater emphasis on psychological support, digital education, and sustained patient engagement remains critical to achieving comprehensive, patient-centered ILD care.

### 5.9. Comparison to Recent Publications

Comparing our findings to previous analyses in IPF or ILD is challenging due to differences in cohort composition, methodology, and the use of varying PROMs and outcome measures. Nonetheless, consistent themes emerge across studies. As in the dataset by Graham et al., our participants described dyspnea, cough, and fatigue as the most burdensome symptoms, often persisting despite antifibrotic therapy [31]. Oxygen therapy was perceived ambivalently—simultaneously a lifeline and a visible marker of illness associated with embarrassment and social withdrawal [8]. European respondents, however, placed greater emphasis on structured rehabilitation and psychosocial support, reflecting the benefit of integrated ILD networks that facilitate access to specialized care but still face challenges in psychological support and long-term continuity [11].

Our results also align with Lancaster et al., who demonstrated persistent diagnostic delays, underrecognition of symptoms by physicians, and reduced quality of life despite therapeutic advances [32]. Similarly, Burnett et al. and Overgaard et al. highlighted ongoing dissatisfaction with communication at diagnosis, treatment tolerability issues, and the emotional and relational strain experienced by both patients and caregivers [33,34]. Together, these studies and our data reveal that while ILD care has advanced pharmacologically, communication quality, coordinated multidisciplinary management, and family-centered psychosocial and palliative support remain critical unmet needs in improving the lived experience of pulmonary fibrosis.

### 5.10. Strengths and Limitations

The principal strength of this study lies in its patient-centered design and its integration of real-world perspectives across multiple dimensions of living with pulmonary fibrosis. By capturing patients’ subjective experiences of mobility, independence, and emotional well-being, the survey extends beyond physiological metrics to illuminate the broader impact of the disease on daily life. The use of validated instruments, including the MRC Dyspnea Scale, VAS-Cough, CFS, and EQ-5D-5L, ensured methodological rigor and facilitated comparison with international datasets. The consistent patterns observed across PROM domains support the validity of our findings and underscore the central role of patient engagement in informing ILD care and research priorities. Importantly, this survey not only applies established PROMs but also uniquely combines frailty assessment with real-world data on mobility limitations, oxygen dependency, and psychosocial distress. This approach highlights clinically relevant, treatable traits that remain insufficiently addressed in ILD.

Limitations include the self-reported nature of data, potential selection bias toward more engaged and health-literate participants, and a modest sample size that limited subgroup analyses. This study did not collect age, comorbidities, or other sociodemographic variables due to the intentional restriction of survey length to reduce participant burden. The exploratory nature of the study, together with retrospective CFS assessments, introduces potential recall bias. Furthermore, medical documentation was optional due to the anonymous survey structure. Nevertheless, the coherence of results across physical, functional, and psychological dimensions supports their external validity.

## 6. Conclusions

Taken together, these findings demonstrate that even within well-structured healthcare systems, people living with pulmonary fibrosis continue to experience profound physical, emotional, and social challenges. While pharmacological advances have extended survival, they have not substantially alleviated the lived burden of disease. Integrating patient-reported outcomes, structured rehabilitation, and psychosocial support into routine ILD care is therefore essential to address the full spectrum of patient needs.

Future initiatives should prioritize longitudinal assessment of PROMs, patient-experience metrics, and cross-national benchmarking to capture what truly matters to patients—preserving mobility, autonomy, and quality of life. The consistent convergence of evidence across continents underscores that symptom relief, functional independence, and psychological well-being remain key unmet needs in ILD care.

A multidimensional approach that combines early antifibrotic initiation with personalized symptom management, comprehensive rehabilitation, and proactive mental health support will be crucial to improving patient outcomes. Moreover, standardized communication frameworks and validated educational resources, co-developed by professional societies and patient organizations, can enhance patient empowerment and ensure consistency of care across regions. Embedding these patient-centered principles into European ILD registries such as eurILDreg will enable continuous, real-world evaluation of quality of life as a central measure of care performance and success.

## Figures and Tables

**Table 1:** Prevalence of supportive aids and interventions in the ILD Cohort.

Device/Support	% of Respondents
Pulmonary Rehabilitation (outpatient setting, i.e., breathing- or physiotherapy)	60.0%
Oxygen at rest	25.7%
Oxygen during exertion	51.4%
Non-invasive ventilation (NIV)	2.9%
Pulse oximeter	48.6%
Rollator	8.6%
Wheelchair	5.7%
None/Other	11.4%

Abbreviations: NIV—Non-invasive ventilation; ILD—Interstitial Lung Diseases; %—Percentage.

## Data Availability

Data is available upon request.

## Supplementary Materials

The following supporting information can be downloaded at: https://www.mdpi.com/article/10.3390/jcm14248697/s1, File S1: Survey Instruments.
