# Supplementary material for: The Patient Journey in Interstitial Lung Disease: Mobility, Independence, and Psychological Burden
Source: J Clin Med. 2025 Dec 8;14(24):8697. doi: 10.3390/jcm14248697 (PMC12733495; doi:10.3390/jcm14248697)
Supplement: Supplementary file 1 [file jcm-14-08697-s001.zip › jcm-4012156-supplementary.pdf]

Patient Survey – Questionnaire

No.	Question	Response Options
**1**	In which country do you currently live?	Free text
**2**	How satisfied are you in general with the medical care related to your pulmonary fibrosis? (Scale 1–10, 1 = not satisfied, 10 = very satisfied)	1–10
**3**	When did your pulmonary-fibrosis–related symptoms begin?	Date (day/month/year)
**4**	When did you receive the diagnosis of pulmonary fibrosis?	Date (day/month/year)
**5**	Are you receiving antifibrotic therapy?	• Yes, Pirfenidone (“Esbriet”)<br>• Yes, Nintedanib (“Ofev”)<br>• No, not tolerated<br>• No, not offered<br>• No, according to physician not necessary
**6**	What type of pulmonary fibrosis do you have?	• Idiopathic pulmonary fibrosis (IPF)<br>• Pulmonary fibrosis related to rheumatic/autoimmune disease<br>• Hypersensitivity Pneumonitis<br>• Sarcoidosis<br>• Not sure<br>• Other ILD
**7**	How many hours per day are you generally mobile (i.e., walking)?	• Less than 1 hour<br>• 1–2 hours<br>• 2–4 hours<br>• 4–6 hours<br>• More than 6 hours
**8**	Do you already need any of the following aids?	• Oxygen during exertion<br>• Oxygen at rest<br>• Rollator<br>• Wheelchair<br>• Non-invasive ventilation (NIV)<br>• Regular respiratory/physiotherapy<br>• Oxygen finger clip (pulse oximetry)<br>• No aids
**9**	On a scale from 1–10, how limited are you in your daily life? (1 = not at all, 10 = very limited)	1–10
**10**	How severe is your breathlessness currently? (MRC Score)	• Breathlessness only with strenuous activity<br>• Breathlessness when walking fast or uphill<br>• Walks slower than people of same age because of breathlessness or needs to stop occasionally

No.	Question	Response Options
		• Needs to stop after walking ~100 m or a few minutes<br>• Too breathless to leave the house or breathless when dressing/undressing
**11**	How severe is your cough TODAY? (0–100 scale; 0 = no cough, 100 = maximal cough)	0–100
**12**	Based on the previously shown scale: How would you describe your current condition (Clinical Frailty Score, CFS 1–9)?<br>How was your condition 1 year ago?<br>How was your condition 5 years ago?	CFS 1–9 for each time point
**13**	We want to know how good or bad your health is TODAY (EQ-5D-5L Visual Analogue Scale 0–100, 100 = best health).	0–100
**14**	EQ-5D-5L: Mobility	• 1 No problems walking around<br>• 2 Slight problems walking around<br>• 3 Moderate problems walking around<br>• 4 Severe problems walking around<br>• 5 Unable to walk around
**15**	EQ-5D-5L: Self-care (washing and dressing)	• 1 No problems washing or dressing myself<br>• 2 Slight problems washing or dressing myself<br>• 3 Moderate problems washing or dressing myself<br>• 4 Severe problems washing or dressing myself<br>• 5 Unable to wash or dress myself
**16**	EQ-5D-5L: Usual activities (work, study, housework, family, leisure)	• 1 No problems with usual activities<br>• 2 Slight problems<br>• 3 Moderate problems<br>• 4 Severe problems<br>• 5 Unable to perform usual activities
**17**	EQ-5D-5L: Pain or discomfort	• 1 No pain or discomfort<br>• 2 Slight pain or discomfort<br>• 3 Moderate pain or discomfort<br>• 4 Severe pain or discomfort<br>• 5 Extreme pain or discomfort
**18**	EQ-5D-5L: Anxiety or depression	• 1 Not anxious or depressed<br>• 2 Slightly anxious or depressed<br>• 3 Moderately anxious or depressed<br>• 4 Very anxious or depressed<br>• 5 Extremely anxious or depressed
